# Effect of Fe Content on Phase Behavior of Sm–Co–Fe Alloys During Solidification and Aging

**DOI:** 10.3390/ma18081854

**Published:** 2025-04-17

**Authors:** Zhi Zhu, Yikun Fang, Wei Wu, Bo Zhao, Meng Zheng, Ming Lei, Jiashuo Zhang

**Affiliations:** 1 Metallurgical Technology Institute, Central Iron and Steel Research Institute Co., Ltd., Beijing 100081, China; zhuzhi_working@163.com (Z.Z.); zhaobo_working@163.com (B.Z.); leiming20212021@163.com (M.L.); 15931957074@163.com (J.Z.); 2 Division of Functional Materials, Central Iron and Steel Research Institute Co., Ltd., Beijing 100081, China; 13241807980@163.com

**Keywords:** Sm–Co–Fe alloys, arc melting, aging

## Abstract

The effect of different Fe contents on the phases of Sm–Co–Fe ternary alloys during solidification is investigated herein by melting the alloys using a non-consumable vacuum arc furnace. In particular, the phases of the Sm_25.5_Co_bal_Fe_x_ (*x* = 19, 21, 23, and 25 wt.%) alloys are investigated after solidification and aging. The results obtained from Cai Li’s modified Miedema model show a strong interaction between the Sm–Co alloy atoms. Additionally, the results obtained from the Toop geometric model show a strong interaction between the Sm–Co–Fe ternary alloy atoms, enabling the formation of intermetallic compounds. The experimental results show that when the Sm content is 25.5 wt.%, the SmCo_5_ phase gradually decreases as the Fe content increases and disappears when the Fe content is 25 wt.%. Thermodynamic calculations show that when the Fe content is 19 wt.%, there is a region where the SmCo_5_ and Sm_2_Co_17_ phases co-exist. As the Fe content increases, this region gradually decreases. For a 25 wt.% Fe content, the Sm_2_Co_17_ and SmCo_5_ two-phase region does not appear when the Sm content varies. The samples are aged at 1143 °C for 12 h, then melted and cut. The phase results obtained by scanning are consistent with the calculated results. In this study, the effect of each constituent element of Sm–Co–Fe ternary alloys on their solidification phases is investigated, providing a strong foundation for studying the 2:17-type Sm–Co magnetic materials obtained after melting and aging a five-element Sm–Co alloy.

## 1. Introduction

Sm_2_Co_17_-type magnets are widely used in the aerospace industry, computation applications, machinery, household appliances, motor engineering, automation technology, and the automotive industry because of their excellent magnetic properties, high Curie temperature, strong corrosion resistance, and good thermal stability, which are highly important in these fields [1,2,3,4,5]. However, due to the rapid rise in the prices of rare-earth metals [6], persistently high cost of cobalt, and increasing demand for high-performance Sm_2_Co_17_-type magnets [7,8,9,10], enhancing the Fe content in 2:17-type Sm–Co permanent magnets has become important.

Sm_2_Co_17_-type magnets are multi-component alloys developed from binary Sm–Co alloys via the partial substitution of Co with Fe, Cu, and Zr [11]. The effect of Fe on the microstructure and magnetic properties of Sm_2_Co_17_-type magnets is complex [12,13]. Additionally, the formation of regions with Sm_2_(Co,Fe)_17_ and SmCo_5_ phases in such magnets is mainly caused by Fe [14]. When the content Fe, which substitutes Co in permanent magnetic materials, is increased, the magnetic field strength (Ms) of the 2:17R main phase can be increased; this, in turn, increases the maximum magnetic energy area (BH)_Max_ of the magnet, thus reducing its cost [15]. However, an excessive Fe content causes the decomposition of large cellular structure organizations and decreases the content of cell walls; additionally, the resulting Fe–Co phase decreases the coercivity and deteriorates the squareness of the magnet’s hysteresis loop [16]. When the Fe content exceeds a certain critical value (~20 wt.%), the formation of a region with a continuous cell-boundary phase (such as a 1:5 phase) is difficult, resulting in a significant deterioration in the energy product of the magnet [17]. Since Sm, Co, and Fe account for over 90 wt.% in Sm_2_Co_17_-type magnets, it is important to investigate the phase formation and microstructure evolution in Sm–Co–Fe ternary alloys to improve the understanding of the behavior of such magnets.

Significant research has been conducted on the phase transition in Sm–Co–Fe ternary alloys. Schneider et al. [18] investigated the phase equilibrium of the transition-metal-rich region in Sm–Co–Fe ternary alloys and found that Fe is infinitely miscible in Sm_2_(Co,Fe)_17_, although the solubility of Fe in SmCo_5_ is limited. Liu et al. [19] determined the phase equilibrium in Sm–Co–Fe ternary alloys at 1073 K, 1273 K, and 1473 K; additionally, they determined the solubility of Fe in SmCo_5_, Sm_5_Co_19_, and Sm_2_Co_7_. Further, Liu et al. [20] calculated the magnetization of Sm(Co_1−x_, Fe_x_)_5_ for *x* = 0–1 using first principles methods; their results showed that the magnetization peaked at 10.6 μB for *x* = 0.6. Fartushna et al. [14] experimentally investigated the phase equilibrium in Co–Fe–Sm alloys; in particular, they investigated the effect of Fe on the intermetallic phase structure and magnetic properties of the alloys and found that the saturation magnetization increased with an increasing Fe content. In the above studies, the phase transition in Sm–Co–Fe ternary alloys under an increasing Fe content was not investigated. Therefore, it is necessary to investigate the phase transition during solidification and aging when the Fe content is high.

The magnetic performance of Sm–Co–Fe ternary alloys deteriorates as the Fe content increases. The mixing enthalpies of corresponding binary and ternary alloys were calculated using Cai Li’s modified Miedema model and the Toop geometrical model. Additionally, the solidification behavior and phase distribution of Sm_25.5_Co_bal_Fe_x_ (*x* = 19, 21, 23, and 25 wt.%) alloys were investigated. Furthermore, thermodynamic equilibrium calculations were performed to investigate changes in regions with Sm_2_(Co,Fe)_17_ and SmCo_5_ phases in the Sm–Co–Fe alloys when the Fe and Sm contents varied. This study lays a solid foundation for improving the understanding of the initial alloy melting process in the preparation of Sm_2_Co_17_-type magnets.

## 2. Materials and Methods

### 2.1. Experimental Procedure

Sm_25.5_Co_bal_Fe_x_ (*x* = 19, 21, 23, and 25 wt.%) alloy samples were prepared using the WS-4 non-consumable vacuum arc furnace (Technical Service Company of the Institute of Physics, Chinese Academy of Sciences). In particular, the samples were placed in a crucible of the furnace chamber and processed. Due to the strong volatility of elemental Sm, an additional 2 wt.% of Sm was added to compensate for its loss during the melting process. Initially, the furnace chamber was vacuumed to a pressure of 5 × 10^−3^ Pa, followed by three cycles of Ar gas filling and purging to clean the environment. The Ar gas was set to a −0.05 MPa pressure, and arc discharge was initiated for the melting process. The weight of each alloy ingot was ~50 g; after each cycle, the ingots were flipped over. This process was repeated four times to ensure a uniform composition. Then, the melted samples were cut, and those with different Fe contents were treated separately. Afterwards, the cut samples were placed in vacuum tubes, evacuated, and sealed using the Ar gas. Finally, after being annealed at 1143 °C for 12 h, the samples were quenched.

### 2.2. Characterization Methods

The phase compositions of the Sm–Co–Fe ternary alloys with different Fe contents were investigated using the MDI-Jade 6.5 software, which was used to fit the X-ray diffraction (XRD) data. The XRD equipment used was a Rigaku D/MAX-2600 diffractometer (Rigaku, Tokyo, Japan) (equipped with a Cu-Kα radiation source) operating at 40 kV/30 mA. The diffraction angle (2θ) scan range was 20–80°, with a step size of 0.01° and a scan time of 1.8 s per step. The diffraction peaks of various phases in the samples were identified using corresponding PDF cards obtained from the Inorganic Crystal Structure Database (ICSD). The samples were analyzed using an electron probe micro-analyzer (EPMA, JXA-8230, JEOL, Tokyo, Japan) for micro-area compositional analysis. The distribution of different phases during the alloy solidification process and the concentrations of various elements within the phases were investigated using scanning electron microscopy (SEM; JSM 7600 F, JEOL Ltd., Tokyo, Japan) equipped with a backscattered electron (BSE) detector. A thermodynamic analysis of the phase diagrams and thermodynamic equilibrium of the Sm–Co–Fe ternary alloys with varying Fe contents was performed using the Thermo-Calc software (Version 3.1, Sm–Co–Fe alloy database [19], Stockholm, Sweden).

### 2.3. Thermodynamic Calculations

Cai Li’s modified Miedema mixing enthalpy model [21], which is a semi-empirical model, is used to describe the mixing enthalpy of binary alloys. The mixing enthalpy of binary alloys can be expressed as follows [22,23]:

(1) $$∆H_{AB}=f_{B}^{A}\frac{x_{A}[1+\mu_{A}x_{B}(\varphi_{A}-\varphi_{B})]\times x_{B}[1+\mu_{B}x_{A}(\varphi_{B}-\varphi_{A})]}{x_{A}V_{A}^{2/3}\left[1+\mu_{A}x_{B}\left(\varphi_{A}-\varphi_{B}\right)\right]+x_{B}V_{B}^{2/3}[1+\mu_{B}x_{A}(\varphi_{B}-\varphi_{A})]}$$

(2) $$f_{B}^{A}=\frac{2PV_{A}^{2/3}V_{B}^{2/3}[\frac{Q}{P}\left(n_{WSA}^{1/3}-n_{WSB}^{1/3}\right)-{{(\varphi }_{A}-\varphi_{B})}^{2}-\alpha \frac{R}{P}]}{{{(n}_{WSA}^{1/3})}^{-1}+{{(n}_{WSB}^{1/3})}^{-1}}$$

(3) $$b\left(x\right)=1-\frac{1}{2}*\frac{x_{A}x_{B}[V_{A}^{2/3}-V_{B}^{2/3}]}{x_{A}^{2}V_{A}^{2/3}+x_{B}^{2}V_{B}^{2/3}}$$

(4) $$∆H_{new}=∆H_{AB}*b(x)$$

where $∆H_{AB}$ is the mixing enthalpy and $f_{B}^{A}$ indicates the proportion at the interface where A atoms are in contact with B atoms, with A atoms considered as the fraction of A atoms that are nearest neighbors. Further, $x_{A}$ and $x_{B}$ are the atomic fractions of components A and B, respectively. $\varphi_{A}\;$and $\varphi_{B}$ are the electronegativity parameters of components A and B, respectively. $n_{WSA}^{1/3}$ and $n_{WSB}^{1/3}$ are the electron densities of components A and B, respectively. $V_{A}^{2/3}$ and $V_{B}^{2/3}$ are the molar volumes of the A- and B-component atoms. Finally, *Q*, *R*, *P*, *μ*, and *α* are empirical parameters. In Equation (2), the *R/P* term is included only when transitioning from a transition to non-transition metals during alloying, and *Q/P* is set to 9.4 (although the value of *P* is different for different alloys). Further, *μ* is a constant that depends on the oxidation state of elements, as follows: (1) *μ* = 0.14 for univalent or alkali metals; (2) *μ* = 0.10 for divalent metals; (3) *μ* = 0.07 for trivalent metals, as well as Cu, Ag, and Au; and (4) *μ* = 0.04 for all other metals. For liquid and solid alloys, *α* is set to 0.73 and 1, respectively.

The thermodynamic properties of ternary alloys can be extrapolated using the Toop geometric model, which is expressed as follows:

(5) $$∆Z_{ABC}^{E}=\frac{x_{B}}{1-x_{A}}∆Z_{AB}^{E}\left(x_{A},\;1-x_{A}\right)+\frac{x_{C}}{1-x_{A}}∆Z_{AC}^{E}\left(x_{A},\;1-x_{A}\right)+{\left(x_{B}+x_{C}\right)}^{2}∆Z_{BC}^{E}(\frac{x_{B}}{x_{B}+x_{C}},\frac{x_{C}}{x_{B}+x_{C}})$$

where $∆Z_{ABC}^{E}$ represents the excess thermodynamic properties of ternary alloys; $∆Z_{AB}^{E}$, $∆Z_{AC}^{E}$, and $∆Z_{BC}^{E}$ represent the excess thermodynamic properties of binary alloys; and $x_{A}$, $x_{B}$, and $x_{C}$ are the molar fractions of components A, B, and C, respectively, in ternary alloys.

## 3. Results and Discussion

The basic physical parameters of Sm, Co, and Fe are presented in Table 1 [24].

**Figure 1:**
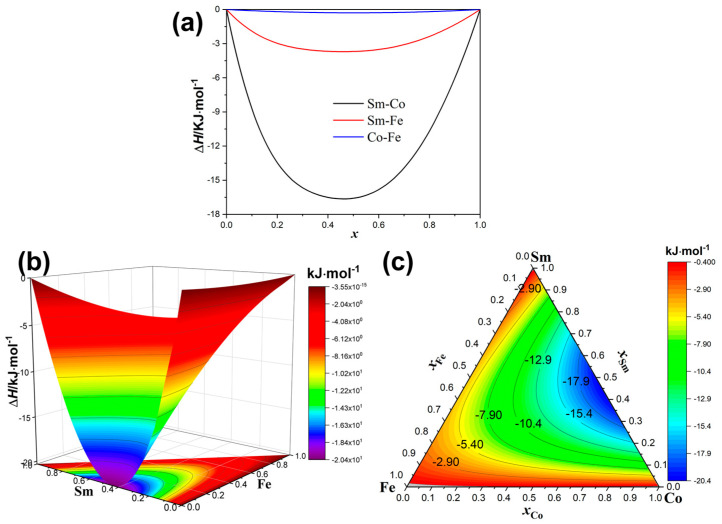
Mixing enthalpies of Sm–Co–Fe ternary alloys: (**a**) mixing enthalpies of the Co–Sm, Fe–Sm, and Co–Fe binary alloys; (**b**) 3D surface and contour plots of the mixing enthalpies of the Sm–Co–Fe ternary alloys; and (**c**) contour plot of the mixing enthalpy of the Sm–Co–Fe ternary alloys.

Figure 1 shows the mixing enthalpies of the binary and ternary alloys of Sm, Co, and Fe. As shown in Figure 1a, the mixing enthalpies of the Co–Fe, Sm–Co, and Sm–Fe binary alloys are all negative, with the mixing enthalpy of Sm–Co being the most negative. This indicates a strong interaction between the Sm and Co atoms, while the interactions between the constituent atoms in Sm–Fe and Co–Fe are weaker than those in Sm–Co. During the alloy melting process, the constituent atoms in the Sm–Co alloy combine preferentially. The *x* and *y* coordinates denote the molar fractions of Sm and Fe, respectively; the sum of the molar fractions of Sm, Co, and Fe in the ternary alloy is one. Therefore, when the molar fractions of Sm and Fe are known, the molar fraction of Co can be uniquely determined. The mixing enthalpy (*ΔH*) of the Sm–Co–Fe ternary alloy is shown in Figure 1b, where *ΔH* is negative over the entire composition range of the ternary alloy. The minimum value of *ΔH* (−15.3139 kJ/mol) is obtained for *x*_Sm_ = 46%. This indicates that in the Sm–Co–Fe ternary alloy, Sm and Co show a higher affinity than Sm and Fe or Co and Fe. As shown in Figure 1c, the three thermodynamic parameters of the Sm–Co–Fe ternary alloy are mainly affected by variations in the molar fraction of Sm, which shows the characteristic of being large at both ends and small in the middle. As the Sm molar fraction increases, the mixing enthalpy of the Sm–Co–Fe ternary alloy initially decreases and then increases, exhibiting an overall parabolic curve. The minimum mixing enthalpy is observed for the Sm–Co alloy, indicating that Sm readily combines with Co to form intermetallic compounds. Furthermore, in the Sm_25.5_Co_bal_Fe_x_ (*x* = 19, 21, 23, and 25 wt.%) ternary alloys, the mixing enthalpy remains below −12.9 kJ/mol. This indicates that the Sm–Co–Fe ternary system can easily form intermetallic compounds. This provides a theoretical foundation for the phase formation in Sm–Co–Fe ternary alloys.

**Figure 2:**
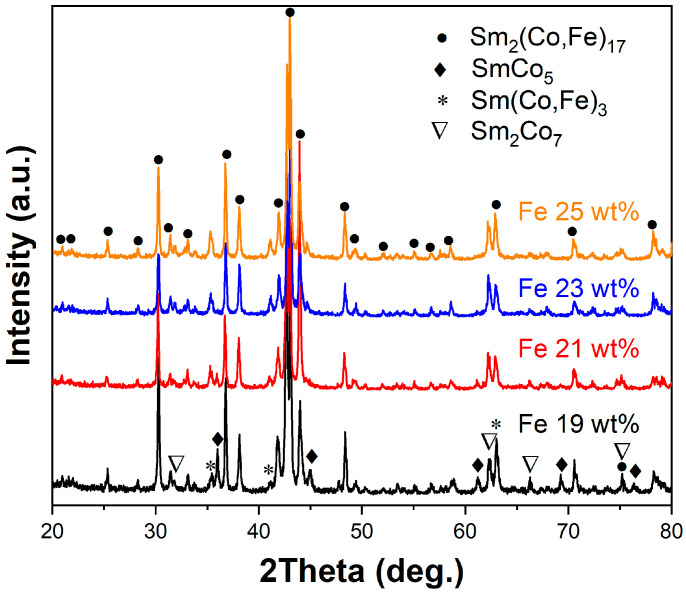
XRD spectra of solidified Sm_25.5_Co_bal_Fe_x_ (*x* = 19, 21, 23, and 25 wt.%) ternary alloys.

Figure 2 shows the XRD patterns of solidified Sm–Co–Fe ternary alloys with different Fe contents (Fe = 19, 21, 23, and 25 wt.%). It is observed that when the Fe content is 19 wt.% (Sm_25.5_Co_74.5_Fe_19_), the alloy is mainly characterized by the presence of Sm_2_(Co,Fe)_17_, SmCo_5_, Sm_2_Co_7_, and Sm(Co,Fe)_3_. The diffraction peak intensities of the Sm_2_(Co,Fe)_17_, SmCo_5_, and Sm_2_Co_7_ phases are relatively high, indicating that these phases occupy a larger proportion in the alloy. In contrast, the diffraction peak intensity of the Sm(Co,Fe)₃ phase is lower, suggesting that this phase has a smaller proportion. Among them, the Sm_2_(Co,Fe)_17_ phase exhibits the highest diffraction intensity, which is greater than that of the SmCo_5_ and Sm_2_Co_7_ phases. As the Fe content increases, the diffraction peak intensity of the Sm_2_(Co,Fe)_17_ phase exhibits an overall increasing trend; Sm_2_Co_7_ and Sm(Co,Fe)_3_ exhibit a similar trend. Conversely, the peak intensity of the SmCo_5_ phase exhibits a decreasing trend. When the Fe content is 25 wt.%, the region with the SmCo_5_ phase disappears and only regions with the Sm_2_(Co,Fe)_17_, Sm_2_Co_7_, and Sm(Co,Fe)_3_ phases are present in the alloy.

**Figure 3:**
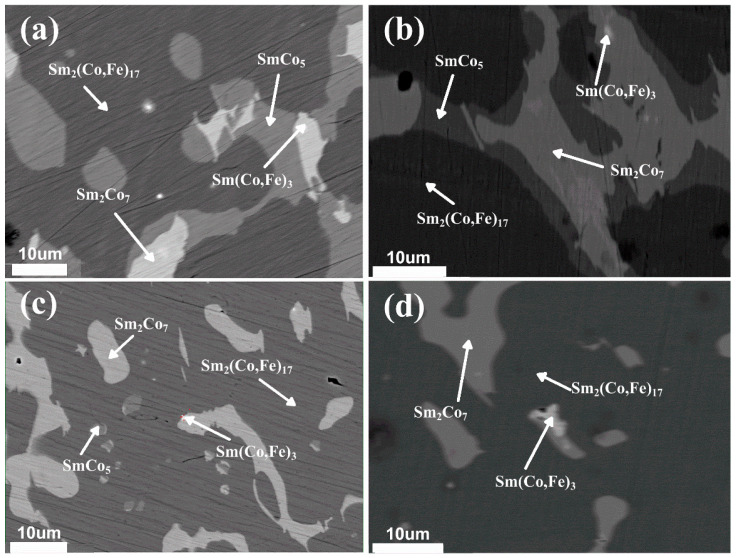
EPMA backscattered images of solidified Sm_25.5_Co_bal_Fe_x_ alloys: (**a**) x = 19 wt.%, (**b**) x = 21 wt.%, (**c**) x = 23 wt.%, and (**d**) x = 25 wt.%.

EPMA backscattered images of the solidified Sm_25.5_Co_bal_Fe_x_ ternary alloys (*x* = 19 wt.%, 21 wt.%, 23 wt.%, and 25 wt.%) are shown in Figure 3. As shown in Figure 3a, when the Fe content is 19 wt.%, the following four main phase regions are observed: a black matrix phase region, a large grey phase region, a white phase region surrounded by the grey phase region, and a bright-white phase region surrounded by the white phase region. The EPMA spot-scanning results are presented in Table 2, where a, b, c, and d correspond to the respective images in Figure 3. The data for each point represent the average value obtained from five spot scans. The XRD results and those presented in Table 2 indicate that the black matrix, grey, white, and bright-white phase regions correspond to Sm_2_(Co,Fe)_17_, SmCo_5_, Sm_2_Co_7_, and Sm(Co,Fe)_3_, respectively. Figure 3b,c show that, as the Fe content increases, the region with the SmCo_5_ phase significantly decreases, whereas the region with the black matrix Sm_2_(Co,Fe)_17_ phase notably increases. When the Fe content is 25 wt.% (Figure 3d), the region with the SmCo_5_ phase disappears and only regions with Sm_2_(Co,Fe)_17_, Sm_2_Co_7_, and Sm(Co,Fe)_3_ phases are observed. These results are consistent with the XRD results.

**Figure 4:**
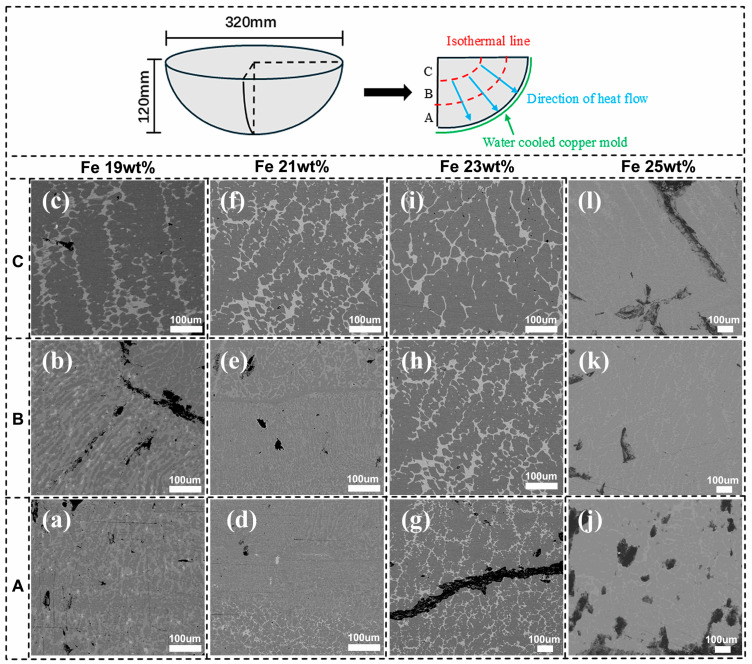
SEM images of the Sm_25.5_Co_bal_Fe_x_ (*x* = 19, 21, 23, and 25 wt.%) ternary alloys: (**a**–**c**) 19 wt.%Fe, (**d**–**f**) 21 wt.%Fe, (**g**–**i**) 23 wt.%Fe, and (**j**–**l**) 25 wt.%Fe. A, B, and C represent different positions of the composition in the ingot.

The shapes and sizes of the ternary alloy samples after the melting process are shown in Figure 4. It is observed that the lower arc surface of each sample is in contact with the water-cooled copper mold, indicating that the heat flow direction during the solidification process is perpendicular to the surface of the copper mold. The solidification process starts at the contact surface and is completed towards the central area. The solidified samples are cut along the dashed lines into four regions (A, B, and C), corresponding to the sections spanning from the region in contact with the copper mold to the region where the solidification process is completed. Specifically, region A represents a portion of the alloy ingot at the bottom (where it contacts the copper crucible), while region C is the farthest from the copper mold. The phases and their regional distribution in each sample ingot from the bottom to the top are analyzed for different Fe contents. The study finds that when the Fe contents are 19 wt.% (Figure 4a–c), 21 wt.% (Figure 4d–f), and 23 wt.% (Figure 4g–i), the cooling rates in region A are high, resulting in a fine grey phase region distributed within the black matrix phase region. The black matrix phase region corresponds to the Sm_2_(Co,Fe)_17_ phase; the grey phase region (corresponding to the SmCo_5_ phase) exhibits a striped distribution along the heat flow direction. A small part of the white phase region (corresponding to the Sm_2_Co_7_ phase) precipitates during solidification. As the cooling rate decreases along the heat flow direction during the solidification of the melted alloy, long dendrites are formed, wherein the main phase region is the white phase region (corresponding to the Sm_2_Co_7_ phase). In addition, the grey phase region (corresponding to the SmCo_5_ phase) decreases significantly. Additionally, a small part of the bright-white phase region (corresponding to the Sm(Co,Fe)_3_ phase), wherein the atomic ratio of Sm to (Co,Fe) is 1:3, is observed in the white phase region (corresponding to the Sm_2_Co_7_ phase). This result is consistent with the EPMA results described prior. When the Fe content is 25 wt.% (Figure 4j–l), the black matrix phase region (corresponding to the Sm_2_(Co,Fe)_17_ phase) is maintained, while the white phase region (corresponding to the Sm_2_Co_7_ phase) increases and exhibits fine dendrites along the heat flow direction. Additionally, a small part of the bright-white phase region (corresponding to the Sm(Co,Fe)_3_ phase) is present in the white phase (Sm_2_Co_7_) region. Compared with the results corresponding to 19 wt.%, 21 wt.%, and 23 wt.% Fe contents, it can be seen with an Fe content of 25 wt.% that the SmCo_5_ phase decreases as the Fe content of the alloys increases, with only a small amount of the SmCo5 phase present at 23 wt.% and the SmCo5 phase disappearing when the Fe content reaches 25 wt.%. This result is consistent with the trend observed in the XRD and EPMA data mentioned above.

**Figure 5:**
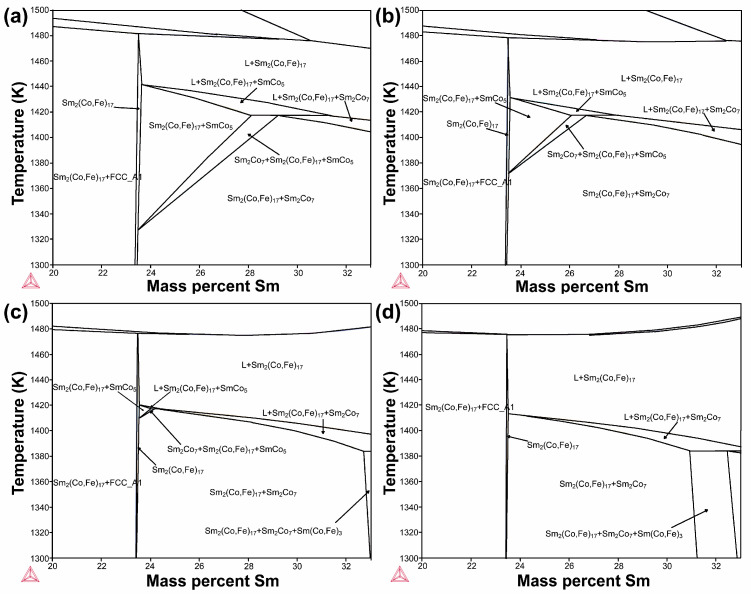
Pseudo-binary phase diagrams of the Sm–Co–Fe ternary alloys: (**a**) 19 wt.%, (**b**) 21 wt.%, (**c**) 23 wt.%, and (**d**) 25 wt.% Fe contents.

Figure 5 shows phase diagrams of the alloys for Sm concentrations in the 20–33 wt.% range for 19, 21, 23, and 25 wt.% Fe contents. As shown in Figure 5a, when the Fe content is 19 wt.%, the Sm concentration in the alloys must be within the 23.45–31.31 wt.% range at a certain temperature to allow for the co-existence of regions with the Sm_2_(Co,Fe)_17_ and SmCo_5_ phases. Additionally, a single region with the Sm_2_(Co,Fe)_17_ phase is observed, while a region with the SmCo_5_ phase is absent. As shown in Figure 5b, it is observed that when the Fe content is 21 wt.%, the Sm concentration in the alloy must be within the 23.42–27.84 wt.% range to allow for the co-existence of the Sm_2_(Co,Fe)_17_ and SmCo_5_ phases. The region where the Sm_2_(Co,Fe)_17_ and SmCo_5_ phases co-exist is significantly smaller than that observed at an Fe content of 19 wt.%. The above results indicate that, as the Fe content increases, the region where the Sm_2_(Co,Fe)_17_ and SmCo_5_ phases co-exist gradually decreases. When the Fe content is 25 wt.%, the region where the Sm_2_(Co,Fe)_17_ and SmCo_5_ phases co-exist disappears. This indicates that when the Fe content is 25 wt%, the 1:5 phase disappears, and changes in the Sm content do not alter this result. Additionally, the single-phase region with the Sm_2_(Co,Fe)_17_ phase gradually decreases as the Fe content increases. To verify the existence of the two-phase region, experiments were conducted to produce these two structural phases via aging at 1143 °C for 12 h to fabricate a magnet.

**Figure 6:**
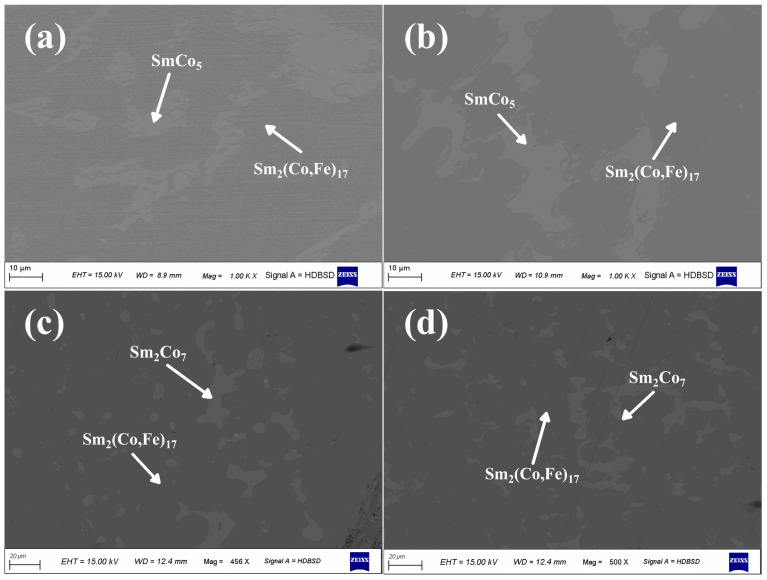
SEM images of the Sm_25.5_Co_ba1_Fe_x_ alloys undergoing aging for 12 h at 1143 °C using (**a**) 19 wt.%, (**b**) 21 wt.%, (**c**) 23 wt.%, and (**d**) 25 wt.% Fe contents.

Figure 6 shows SEM images of the Sm–Co–Fe ternary alloys for different Fe contents after aging for 12 h at 1143 °C. As shown in Figure 6a, only two regions (corresponding to the Sm_2_(Co,Fe)_17_ and SmCo_5_ phases) were observed when the Sm and Fe contents were 25.5 wt.% and 19 wt.%, respectively. As shown in Figure 6b, the same results were observed when the Fe content was 21 wt.%; additionally, the region with the SmCo_5_ phase decreased as the Fe content increased. As shown in Figure 6c, regions with the Sm_2_(Co,Fe)_17_ and Sm_2_Co_7_ phases were observed after aging when the Fe content was 23 wt.%, and no SmCo_5_ phase was present. The presence of the SmCo_5_ phase in the solidified samples before aging was mainly due to the rapid cooling during the cooling process of the alloy liquid, resulting in significant undercooling, which is a non-equilibrium solidification process. The alloy with an Fe content of 23 wt.% crossed the SmCo_5_ phase, leading to the existence of the SmCo_5_ phase. As shown in Figure 6d, the results obtained for an Fe content of 25 wt.% after aging were consistent with those observed for an Fe content of 23 wt.%. This observation corroborates the thermodynamic phase diagram calculations shown in Figure 5.

## 4. Conclusions

In this study, Sm–Co–Fe ternary alloys with different Fe contents were prepared using a non-consumable vacuum arc furnace. Further, their phase changes during solidification and aging processes were investigated by varying the Fe content. The results of this study are summarized below.

Cai Li’s modified Miedema model was combined with the Toop geometric model and used to calculate the mixing enthalpies of liquid Sm–Co–Fe alloys. For the Sm_25.5_Co_bal_Fe_x_ (*x* = 19, 21, 23, and 25 wt.%) alloys, the mixing enthalpy was below −12.9 kJ/mol, indicating strong atomic interactions and a tendency for intermetallic compound formation.

Solidification experiments showed that when the Sm content and Fe contents were 25.5 wt.% and 19 wt.%, respectively, Sm_2_(Co,Fe)_17_, SmCo_5_, Sm_2_Co_7_ and Sm(Co,Fe)_3_ phases were predominantly present in the alloys. When the Fe content increased, the region corresponding to the SmCo_5_ phase gradually decreased; additionally, when the Fe content was 25 wt.%, the region corresponding to the SmCo_5_ phase disappeared.

The Thermo-Calc software results indicated that the region where the Sm_2_(Co,Fe)_17_ and SmCo_5_ phases co-existed decreased when the Fe content increased; additionally, when the Fe content was 25 wt.%, this region disappeared. These results agreed with the results obtained using alloy samples with different Fe contents after aging.

The SmCo_5_ phase existed when the Fe content was 23 wt.% under solidification in a water-cooled copper mold, mainly due to the rapid cooling of the liquid, which caused it to pass through the SmCo_5_ phase region, resulting in the presence of the SmCo_5_ phase. This was a non-equilibrium solidification process. After aging, the composition reached equilibrium, and the SmCo_5_ phase disappeared.

## Figures and Tables

**Table 1:** The specific related parameters of elements Sm, Co, and Fe.

Element	$n_{ws}^{1/3}$/*d.u.*	*φ*/*V*	$V^{2/3}/\mathrm{cm}$	*μ*	*r*/*p*	*Tm*/*K*
Sm	1.21	3.2	7.37	0.7	0.7	1345
Co	1.75	5.1	3.55	0.04	1	1768
Fe	1.77	4.97	3.69	0.04	1	1811

**Table 2:** Solidification phase and element content of Sm-Co-Fe alloy.

Nominal Composition (wt.%)		Measured Composition by EPMA(at.%)			Identified Phases by EPMA
		Sm	Co	Fe	
a	Sm_25.5_Co_55.5_Fe_19_	9.6	64.4	26	Sm_2_(Co,Fe)_17_
		14.8	64.5	20.7	SmCo_5_
		19.3	63.2	17.5	Sm_2_Co_7_
		23.96	56.44	19.6	Sm(Co,Fe)_3_
b	Sm_25.5_Co_53.5_Fe_21_	9.4	60.27	30.33	Sm_2_(Co,Fe)_17_
		13.67	61.2	25.13	SmCo_5_
		20.6	60.1	19.3	Sm_2_Co_7_
		24.2	57.79	18.01	Sm(Co,Fe)_3_
c	Sm_25.5_Co_51.5_Fe_23_	9.44	61.37	29.19	Sm_2_(Co,Fe)_17_
		13.38	63.6	23.02	SmCo_5_
		19.57	60.56	19.87	Sm_2_Co_7_
		23.39	58.59	18.02	Sm(Co,Fe)_3_
d	Sm_25.5_Co_49.5_Fe_25_	9.47	56.9	33.63	Sm_2_(Co,Fe)_17_
		14.5	57.47	28.03	Sm_2_Co_7_
		24.62	55.08	20.3	Sm(Co,Fe)_3_

## Data Availability

The original contributions presented in this study are included in the article. Further inquiries can be directed to the corresponding authors.
